# Liquid Biopsy in Endometriosis: A Systematic Review

**DOI:** 10.3390/ijms24076116

**Published:** 2023-03-24

**Authors:** Carlo Ronsini, Pietro Fumiento, Irene Iavarone, Pier Francesco Greco, Luigi Cobellis, Pasquale De Franciscis

**Affiliations:** 1 Department of Woman, Child and General and Specialized Surgery, University of Campania “Luigi Vanvitelli”, 80138 Naples, Italy; 2 Unit of Gynecologic Oncology, Department of Woman, Child and Public Health, A. Gemelli, IRCCS, University Hospital Foundation, 00168 Rome, Italy

**Keywords:** endometriosis, micro-RNA, exosomes, cell-derived microparticles, early-stage

## Abstract

Despite laparoscopy being a standardized option to diagnose pelvic endometriotic implants, non-invasive biomarkers are necessary to avoid the discomfort of invasive procedures. Recent evidence suggests a potential role of microRNAs (miRNAs) as feasible biomarkers for the early diagnosis of endometriosis. Following the recommendations in the Preferred Reporting Items for Systematic Reviews and Meta-Analyses (PRISMA) statement, we systematically searched PubMed, EMBASE, Scopus, Cochrane Library, and Science Direct in January 2023. We provided no restriction on the country and year of publication, and considered English published articles. We selected studies including patients with endometriosis and describing miRNA regulation in the context of endometriosis. Overall, 45 studies fulfilled the inclusion criteria, and 2045 patients with endometriosis and 1587 controls were screened. Patients were analyzed concerning miRNAs expression and sources, stage of disease, and symptoms, and compared to controls. Among DEMs, the ones with the widest delta between endometriosis patients and controls—Relative Expression ≥ 4 Log2(ratio)—were miR-145, miR-191, miR-195, miR-21-5p, miR-106b-5p, miR-195-5p, miR-451a, miR-200c, miR-20a-5p, and miR-15a-5p. Although the epigenetic regulation is partially unclear, miRNAs are valid biomarkers to diagnose endometriotic lesions in symptomatic and non-symptomatic women. MiRNAs modulation should be clarified, especially during therapies or relapse, to plan targeted management protocols.

## 1. Introduction

Endometriosis diagnosis in childbearing-age women is often delayed due to the lack of pathognomonic signs and symptoms [1,2,3]. Nowadays, transvaginal ultrasound (TVS) is the most cost-effective method to detect endometriotic lesions, but the gold-standard methodology for diagnosis is laparoscopy [4]. Laparoscopy is also considered the gold-standard treatment for endometriosis [2], while the best treatment option should consider the age of the patient, the symptoms, the desire to conceive, and previous surgeries [4]. In older women who underwent previous surgeries, medical treatment and In Vitro Fertilization may be discussed [4]. That emphasizes the necessity of non-invasive biomarkers to avoid the discomfort of laparoscopic procedures and simplify the diagnosis. Recent studies suggest the possibility of using microRNAs (miRNAs)—as reliable markers from different compartments (serum, plasma, endometrial biopsies, etc.)—in the early diagnosis of endometriosis [5,6,7]. Minimally invasive methods are necessary to assess the influence of bio-behavioral disruptors on the prognosis, treatment response, and recurrence.

The miRNAs are a class of small RNA molecules, composed of 15–22 nucleotides each, post-transcriptionally regulating genes [8]. MiRNAs hybridize into complementary mRNAs, which are involved in different cellular features, such as implantation, embryo developmental processes, tumor suppression, apoptosis, proliferation, angiogenesis, and metastasization [9,10,11]. Despite endometriosis showing a benign histological connotation, it develops and disseminates as a neoplastic process [12,13]. Moreover, there are pieces of evidence that endometriotic implants could transform into cancerous lesions [14]. In that context, endometriosis pathogenesis contains genetic, angiogenic, metabolic, and immunological alterations [12]. Endometriotic implants could undergo malignant transformation via altered molecular pathways, showing characteristics of atypia, invasivity, and diffusion, especially in E-cadherin-negative endometriotic cells [12]. Moreover, in 30% of cases, an endometriosis diagnosis may be linked to ovarian cancer detection [12]. Indeed, there is evidence that miRNAs are also involved in the pathogenesis of ovarian cancer, even in presentations linked to endometriosis [15,16,17]. One of the advantages of miRNAs is that they are accessible to sample. They could be found in multiple cellular compartments—in the context of different human systems—and be up- or downregulated [18,19,20]. It is estimated that miRNAs could be feasible biomarkers in the early diagnosis and management of endometriosis progression [20]. For example, endometriotic lesion development may depend on lower-expression cell adhesion and cytoskeleton molecules and decreased proteolysis [21,22,23]. In those contexts, the proliferation, migration, and stemness of endometrial stromal cells (ESCs) are increased in endometriosis through miRNAs’ epigenetic transcription [24]. Most miRNAs can be found in the serum of endometriosis-affected patients, and they could be extracted through liquid biopsy [15,21]. Those data may pave the way for new strategies for early diagnosis of endometriotic implants. The present systematic review aimed to evaluate the distribution and regulation of the differently expressed miRNAs (DEMs) in the context of endometriosis.

## 2. Materials and Methods

The methods for this study were specified a priori based on the recommendations in the Preferred Reporting Items for Systematic Reviews and Meta-Analyses (PRISMA) statement [25]. The present work is categorized on PROSPERO as: ID400389.

### 2.1. Search Method

We performed a systematic search for records about the expression of different miRNAs in endometriosis-affected patients in PubMed, EMBASE, Scopus, Cochrane Library, and Science Direct in January 2023. We made no restriction on the country or year of publication, and considered only studies published entirely in English. We adopted the following string of keywords in each database to identify studies that fit to the topic of our review: “(Cell-Derived Microparticles OR MicroRNAs) AND Endometriosis”.

### 2.2. Study Selection

The study selection was made independently by I.I. and P.F. In the case of a discrepancy, C.R. decided on inclusion or exclusion. Inclusion criteria were (1) studies including patients with endometriosis; (2) studies describing differently expressed miRNAs (DEMs) and their regulation in the context of endometriosis signs and symptoms; and (3) peer-reviewed articles, published originally. We excluded non-original studies, pre-clinical trials, animal trials, abstract-only publications, and articles in a language other than English. If possible, we tried to contact the authors of studies that were published as conference abstracts via e-mail and asked them to provide their data. We assessed all included studies concerning potential conflicts of interest.

### 2.3. Extraction and Quantification of miRNAs

Liquid biopsy is a minimally invasive procedure to extract microvesicles from serum [12]. Extraction, amplification, and quantization of miRNAs are based on different procedures.

#### 2.3.1. Extracellular Vesicles Classification

Extracellular vesicles (EVs) are a class of various submicron vesicles that can be released by cells in different conditions [26,27,28,29,30,31,32,33,34]. EVs are classified into exosomes, microvesicles, and apoptotic bodies. Exosomes measure from 30 to 100 nm, and they are formed into endosomes. Microvesicles measure from 100 to 1000 nm, and they derive from the plasma membrane, whereas apoptotic bodies measure 0.1–5 μm [35]. In particular, exosomes derived from multivesicular bodies (MVBs), and the “endosomal sorting complex required for transport” (ESCRT) protein complex may regulate their release [36]. Secondarily, MVBs can fuse with the plasma membrane, releasing exosomes. Immunoelectron microscopy revealed tetraspanins CD9, CD63, and CD81 as key components of exosomes, which could be used as biomarkers [37,38,39,40,41,42,43,44], whereas apoptotic bodies are positive for caspases 3 and 7 [45]. Otherwise, EVs have recently been identified according to their dimensions as small, if less than 100 nm, and medium and/or large, when 100–200 nm [46].

#### 2.3.2. Extracellular Vesicles Analysis

EVs are usually recognized through immunoblotting, detecting the presence of tetraspanins in samples [46,47]. Moreover, the transmission electron microscope (TEM) and scanning electron microscope (SEM) assess EVs’ dimensions [48,49,50,51,52], whereas EVs features like elasticity are tested by an atomic force microscope (AFM) [53,54,55]. Flow cytometry (FC) is the most feasible method to analyze EVs’ content [56,57,58,59]. An immunophenotypic assessment may be performed through polychromatic FC [44,60,61,62], whereas FC with fluorescence images guarantees a sensitive method for EVs analysis [63,64].

Other authors described miRNA isolation in endometrial stromal cells from biopsies of ectopic endometrial lesions or eutopic endometria, which were placed into two halves in buffered formaline for storage and molecular examination. The RNA quality was first evaluated according to the integrity of the strains in the samples, whereas further analysis was performed based on the RNA minimum degradation in each sample [65].

Among the DEMs isolated, only those with AUC (Area Under the Curve) > 0.6 and significant allele and genotype distribution frequencies (*p* < 0.05) were considered in the present study.

## 3. Results

### 3.1. Studies’ Characteristics

**Figure 1:**
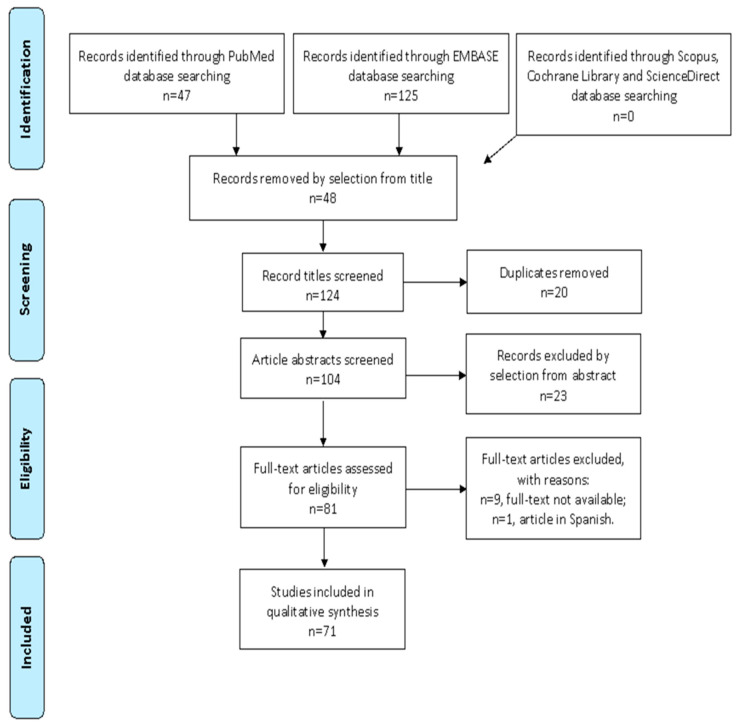
PRISMA (Preferred Reporting Items for Systematic Reviews and Meta-Analyses) Flow-chart.

We mention the studies selected and all reasons for exclusion in the Preferred Reporting Items for Systematic Reviews and Meta-Analyses (PRISMA) flowchart (Figure 1). After the database search, 124 articles matched the search criteria. After removing records without full text, duplicates, and wrong study designs (e.g., reviews), 64 were eligible. Overall, 45 matched the inclusion criteria and were included in the systematic review. The countries where the studies were conducted, the year range, the studies’ design, and the number of participants are summarized in Table 1. Overall, the publication years ranged from 2013 to 2022. In total, 2045 patients with endometriosis and 1587 controls were analyzed.

### 3.2. Outcomes

A total of 2045 patients were included in the review. Regarding miRNA sources, miRNAs in endometrial stromal cells (ESCs) were extracted from the biopsies of ectopic endometrial lesions and/or eutopic endometria. Otherwise, miRNAs were extracted from serum, plasma, follicular fluid, and cumulus cells. Those data are summarized in Table 2 and Table 3.

#### 3.2.1. Early Diagnosis

In total, 58 miRNAs were upregulated in endometriosis-affected patients, whereas, 67 miRNAs were downregulated. Those data are summarized in Table 2 and Table 3. Except for 8, the other 37 studies revealed the stage of disease. In total, 18 records involved patients with ASRM (American Society for Reproductive Medicine) stages III–IV of disease; 16 records involved patients with ASRM stages I–IV of disease; whereas only 3 studies included patients with low or intermediate stages of disease (I–III). In particular, Wang et al. enrolled 30 patients with stages I–II of disease and revealed upregulation of miR-20a-5p through liquid biopsy [71]. In parallel, liquid biopsy showed downregulation of miR-30c-5p, miR127-3p, miR-99b-5p, and miR-15b-5p in the same cohort [71]. Liu et al. isolated miRNAs from ESCs, both in eutopic and ectopic endometria, demonstrating that miR-449b-3p was downregulated in the early stages of endometriosis-affected women [74]. Petracco et al. enrolled patients with stages II–III of endometriosis [80]. MiRNA was isolated both from eutopic and ectopic endometrial samples, and miR-135a/b was downregulated [80]. In the last two studies, the difference in the relative expression of miRNAs between patients and controls was <2 Log2(ratio) [74,80].

#### 3.2.2. Early Diagnosis

Pokrovenko et al. enrolled 64 endometriotic infertile patients with dysmenorrhea in stages I–IV of disease, and miRNA extraction revealed downregulation of miR-let-7 [94], although the relative expression in the Log2(ratio) between the patients and controls corresponded to 0.6 [94]. Regarding dysmenorrhea, in the Bendifallah et al. study, 100% of the patients with stages I–IV of endometriosis were dysmenorrheic [99]. In particular, liquid biopsy demonstrated that hsa-miR-29b-1-5p, hsa-miR-4748, hsa-miR-515-5p, hsa-miR-548j-5p, and hsa-miR-6502-5p were upregulated, whereas hsa-miR-3137 and hsa-miR-3168 were downregulated, with no specification of the relative expression pattern [99]. Both endometrial biopsies and plasma showed that miR-124-3p was downregulated in the Dabi et al. analysis, even though the authors did not declare the stage of disease of the patients enrolled [100]. Regarding infertility, Xu et al. enrolled 14 infertile endometriotic patients, with no specification of the stage of disease, in whose endometrial biopsies miR-1304-3p, miR-544b, miR-3684, miR-494-5p, miR-4683, and miR-6747-3p were upregulated, whereas miR-3935, miR-4427, miR-652-5p and miR-205-5p were downregulated [73]. In the da Silva et al. study, 100% of the patients with stages I–IV of disease were infertile, and miRNA extraction from cumulus cells revealed downregulation of miR-532-3p [84]. Only Li et al. analyzed the follicular fluid of infertile patients with stages III–IV of disease, revealing downregulation of miR-451 [78]. Those results are summarized in Table 4.

#### 3.2.3. MiRNAs Relative Expression in Patients and Controls

Among DEMs, the ones with the widest delta between endometriosis patients and controls—Relative Expression ≥ 4 Log2(ratio)—were miR-145, miR-191, miR-195, miR-21-5p, miR-106b-5p, miR-195-5p, miR-451a, miR-200c, miR-20a-5p, and miR-15a-5p [21,68,69,72,83,88,96]. In parallel, DEMs with an intermediate delta between patients and controls—Relative Expression ≥ 2 > 4 Log2(ratio)—were miR-146a rs2910164, miR-149 rs2292832, miR-16, miR-29c-3p, miR-451, miR-10b, miR-199a-3p, miR-205-5p/ZEB1, miR-519b-3p/PRRG4, and miR-423 rs6505162 [20,68,69,78,83,88,95,98,104]. For example, Borisov et al. highlighted the widest difference in the relative expression of upregulated miR-191 between endometriotic patients and controls [83]. It was isolated in ESCs from the biopsies of eutopic and ectopic endometria [83]. Afterwards, in the Braza-Boïls et al. study, we found that miR-21-5p was the second most upregulated miRNA in endometriotic patients compared to controls [68]. miRNA was extracted from eutopic endometria in that case also [68]. Secondarily, upregulated miR-145 and miR-451a show a difference ≥ 4 Log2(ratio) in relative expression between patients and controls (6.5 and 5.2, respectively), and they are extracted from plasma and serum, respectively [21,72]. Those results are summarized in Table 4.

## 4. Discussion

From a functional perspective, miRNAs are involved in intercellular crosstalk, both in eutopic endometrial tissue and endometriotic implants [70]. Scientific literature highlighted the potential role of DEMs as biomarkers for endometriosis-affected women. The expression and modulation of miRNAs are wide and heterogeneous, and we considered in our study only DEMs with the highest AUC (>0.6) and significant allele and genotype distribution frequencies (*p* < 0.05). Hypothetically, miRNAs may indirectly represent the cellular microenvironment that leads to the formation of endometriotic implants. Therefore, their research could help intercept endometriosis before macroscopic lesions are identifiable on an ultrasound. Although it is extremely difficult to determine the most sensitive and specific miRNAs in endometriosis pathogenesis, we have underlined miRNAs expression in symptomatic patients. For example, specific miRNAs are overexpressed in dysmenorrheic or infertile women. The presence of the symptom can help us in a twofold way. It can help us identify patients for further investigation by liquid biopsy. It can also give us information about how patients evolve to this symptomatology, helping us to understand the molecular mechanisms underlying the development of the symptomatology. This consideration is also interesting from the perspective of infertility symptoms without organic pelvic distorting lesions. In these cases, infertility is likely related to the uterine microenvironment corrupted by a chronic inflammatory state. The study of miRNAs in these patients can identify conditions invisible to the eye. Farsimadan et al. isolated miRNAs in infertile endometriotic patients without any declared symptoms, and they highlighted the upregulation of miR-146a rs2910164 and miR-149 rs2292832, which showed an intermediate delta of relative expression between patients and controls, e.g., ≥2 > 4 Log2(ratio) [20]. Further, Li et al. isolated miR-451 as upregulated miRNA in infertile endometriotic patients, but they did not declare whether those patients were suffering from other symptoms, such as dysmenorrhea [78]. However, their analysis revealed an intermediate difference in miR-451 relative expression between patients and controls [78]. On the other hand, there is little evidence about miRNA expression in endometriotic patients suffering from dysmenorrhea, but without infertility-related problems. Bendifallah et al. showed upregulation of hsa-miR-29b-1-5p, hsa-miR-4748, hsa-miR-515-5p, hsa-miR-548j-5p and hsa-miR-6502-5p, whereas Dabi et al. revealed upregulated miR-124-3p, even though neither of the studies specified the incidence of infertility in their cohorts [99,100]. Moreover, neither of the studies disclosed the difference in the relative expression of miRNAs between patients and controls [99,100]. Farsimadan et al. isolated miRNAs in infertile endometriotic patients without any declared symptoms, and they highlighted the upregulation of miR-146a rs2910164 and miR-149 rs2292832, which showed an intermediate delta of relative expression between patients and controls, e.g., ≥2 > 4 Log2(ratio) [20]. Further, Li et al. isolated miR-451 as an upregulated miRNA in infertile endometriotic patients, but they did not declare whether those patients were suffering from other symptoms, such as dysmenorrhea [78]. However, their analysis revealed an intermediate difference in miR-451 relative expression between patients and controls [78]. In our opinion, given the inaccurate definition of the signs and symptoms in different studies—mainly due to the heterogeneity of endometriosis presentation—it would be appropriate to focus on DEMs with the widest range in relative expression between patients and controls to avoid high false-positive rates during miRNA isolation. The real clinical use of miRNAs should lie in implementing the diagnostic capabilities of early forms. With this in mind, the different ways in which miRNA assays can be obtained should be emphasized. The site of expression probably influences miRNA modulation. Liquid and incisional endometrial biopsy may be valid options for miRNA extraction, even if eutopic endometrial tissue seems to have more defined expression profiles [68,83]. Surely, liquid biopsy through patients’ serum or plasma would be more feasible and cost-effective as a screening method in women suffering from dysmenorrhea, infertility, and dyschezia. An endometrial tissue biopsy may be a valid option in diagnosing endometriotic implants in patients with a suspected transvaginal ultrasound. Moreover, serum and plasma miRNAs are supposed to have a distant effect involving systemic organs. That suggests a potential role of liquid biopsy in detecting endometriotic implants of unknown location [20,106]. Although isolation strategies from saliva also revealed positive biomarkers for endometriosis detection, liquid biopsy could find a place in the application of screening programs on the fertile population, even in the complete absence of symptoms [106,107]. Unfortunately, this perspective needs more investigation of miRNA fluctuations in the early stages. For those reasons, one of the best candidates for early diagnosis of endometriosis may be miR-145 [21]. In their study, Bashti et al. showed an important advantage of miR-145—neither of the patients enrolled suffered from infertility or dysmenorrhea [21]. In addition, miR-145 expression is mostly upregulated in stages I–II of disease, suggesting its crucial role in tracking recent endometriotic lesions [21]. Finally, a hypothetical function of miRNAs could be related to the follow-up of patients on medical therapy. Any fluctuations could directly represent the response of endometriosis tissue to medical therapy, optimizing the chronification of treatment. Our review investigated all present literature on the topic. This represents its strength and weakness, related to the enormous heterogeneity of the data in the literature. The hope is that it will represent the basis for further investigation of the most interesting miRNAs useful for the clinical management of patients with endometriosis, with targeted studies specifically designed to investigate the various aspects.

## 5. Conclusions

Most recent evidence shows that intercellular crosstalk has a critical role in endometriosis pathogenesis, although there is heterogeneity of the data. In that context, specific molecular signatures could mark the homeostasis of endometrial tissue. Focusing on endometrial tissue, a pattern of miRNAs may be useful for the early diagnosis and management of endometriosis-affected women, mainly through liquid biopsy. Although there is a lack of data regarding DEMs in response to different therapeutic regimens, that analysis could be performed in women, administered with laparoscopy, oral contraceptives, Non-Steroidal Anti-Inflammatory Drugs (NSAIDs), or a GnRH antagonist during the FU period. To date, although the mechanism of epigenetic regulation remains unclear, the assessment of miRNAs’ expression could be a promising and cost-effective tool to detect the presence of endometriotic implants in symptomatic and non-symptomatic patients. Further studies are needed to clarify miRNAs’ modulation during treatment or the recurrence of disease in order to predict disease development and plan targeted management options.

## Figures and Tables

**Table 1:** Characteristics of included studies.

Author, Year of Publication	Country	Period of Enrollment	Study Type	No. of Patients	No. of Controls
Laudanski 2013 [65]	Poland	N/A	Monocenter prospective case-control	21	25
Jia 2013 [6]	China	2012	Monocenter prospective case-control	23	23
Wang 2013 [66]	China	2011	Monocenter prospective case-control	60	25
Suryawanshi 2014 [67]	USA	2006–2011	Monocenter prospective case-control	33	20
Braza-Boïls 2015 [68]	Spain	N/A	Multicenter prospective case-control	8	11
Cho 2015 [69]	South Korea	2010–2013	Monocenter prospective case-control	24	24
Rekker 2015 [7]	Spain	2010–2014	Multicenter prospective case-control	61	65
Cosar 2016 [70]	USA	2010–2013	Multicenter prospective case-control	24	24
Wang 2016 [71]	China	2011–2013	Monocenter prospective case-control	30	20
Nothnick 2017 [72]	USA	N/A	Multicenter prospective case-control	41	40
Xu 2017 [73]	China	2015	Monocenter prospective case-control	14	10
Bashti 2018 [21]	Iran	N/A	Monocenter prospective case-control	55	23
Liu 2018 [74]	China	2017	Monocenter prospective case-control	19	35
Maged 2018 [75]	Egypt	2015–2016	Monocenter prospective case-control	45	35
Pateisky 2018 [76]	Austria Germany	2010–2012	Monocenter prospective case-control	51	41
Wang 2018 [77]	China	2016–2017	Monocenter prospective case-control	80	60
Li 2019 [78]	China	N/A	Prospective case-control	30	30
Nabiel 2019 [79]	Egypt	2017–2019	Monocenter prospective case-control	51	51
Petracco 2019 [80]	Brazil	2013–2014	Monocenter prospective cohort	23	0
Vanhie 2019 [81]	Belgium	N/A	Monocenter prospective case-control	82	38
Zhang 2019 [82]	China	N/A	Monocenter prospective case-control	10	10
Borisov 2020 [83]	Russia	N/A	Monocenter prospective case-control	10	10
Da Silva 2020 [84]	Brazil	2014–2014	Monocenter prospective case-control	40	13
Gu 2020 [85]	China	N/A	Monocenter prospective case-control	10	10
Mai 2020 [86]	China	N/A	Monocenter prospective case-control	18	10
Moustafa 2020 [87]	USA	2016–2017	Monocenter prospective case-control	41	59
Papari 2020 [88]	Canada	N/A	Monocenter prospective case-control	25	28
Hossein Razi 2020 [89]	Iran	2018–2019	Monocenter prospective case-control	25	25
Wu 2020 [90]	China	N/A	Monocenter prospective case-control	10	10
Zhang 2020 [91]	China	N/A	Monocenter prospective case-control	27	31
Cui 2021 [92]	China	2018–2019	Monocenter prospective case-control	6	6
Farsimadan 2021 [20]	Iran	N/A	Monocenter prospective case-control	260	260
Misir 2021 [93]	Turkey	2017–2018	Monocenter prospective case-control	58	60
Pokrovenko 2021 [94]	Ukraine	N/A	Prospective case-control	64	24
Wang 2021 [95]	China	2019	Monocenter prospective case-control	30	24
Wu 2021 [96]	China	N/A	Prospective case-control	6	3
Zafari 2021 [97]	Iran	N/A	Monocenter prospective case-control	25	25
Bao 2022 [98]	China	2020	Monocenter prospective case-control	20	10
Bendifallah 2022 [99]	France	2021	Prospective case-control	153	47
Dabi 2022 [100]	France	2021	Prospective case-control	153	47
He 2022 [101]	China	N/A	Monocenter prospective case-control	23	20
Huang 2022 [102]	China	N/A	Monocenter prospective case-control	N/A	N/A
Iurova 2022 [103]	Russia	N/A	Monocenter prospective cross-sectional	6	5
Jaafar 2022 [104]	Iraq	N/A	Monocenter prospective case-control	220	220
Nai 2022 [105]	China	2019	Monocenter prospective case-control	30	30

N/A: not available.

**Table 2:** Upregulated microRNA expression profiles in patients with endometriosis.

Author, Year of Publication	MicroRNAs	Source	ASRM Stage of Disease	Regulation in Endometriosis
Wang 2013 [66]	miR-199a miR-122	Serum	III–IV	Up
Suryawanshi 2014 [67]	miR-16 miR-191 miR-195	Plasma	N/A	Up
Braza-Boïls 2015 [68]	miR-16-5p miR-21-5p miR-29c-3p miR-106b-5p miR-130a-5p miR-185-5p miR-195-5p miR-424-5p	Endometrial stromal cells Ectopic endometrium Eutopic endometrium	III–IV	Up
Cosar 2016 [70]	miR-125b-5p miR-451a	Serum	III–IV	Up
Wang 2016 [71]	miR-20a-5p	Serum	I–II	Up
Nothnick 2017 [72]	miR-451a	Serum	I–IV	Up
Xu 2017 [73]	miR-1304-3p miR-544b miR-3684 miR-494-5p miR-4683 miR-6747-3p	Eutopic endometrium	N/A	Up
Bashti 2018 [21]	miR-145	Plasma	I–IV	Up
Maged 2018 [75]	miR-199 miR-122	Serum	I–IV	Up
Pateisky 2018 [76]	miR-33a-5p	Serum	I–IV	Up
Nabiel 2019 [79]	miR-17-5p	Eutopic endometrium	I–IV	Up
Vanhie 2019 [81]	miR-125b-5p miR-28-5p miR-29a3p	Serum	I–IV	Up
Borisov 2020 [83]	miR-191	Eutopic endometrium	N/A	Up
Mai 2020 [86]	miR-506-5p	Serum	III–IV	Up
Moustafa 2020 [87]	miR125b-5p miR-150-5p miR-342-3p miR-451a	Ectopic endometrium	I–IV	Up
Hossein Razi 2020 [89]	miR-185-5p	Plasma	III–IV	Up
Wu 2020 [90]	miR-423-5p	Endometrial stromal cells Ectopic endometrium Eutopic endometrium	III–IV	Up
Zhang 2020 [91]	miR-202-3p	Endometrial stromal cells Ectopic endometrium Eutopic endometrium	III–IV	Up
Farsimadan 2021 [20]	miR-146a rs2910164 miR-149 rs2292832	Serum	N/A	Up
Zafari 2021 [97]	miR-199b-3p	Serum	I–IV	Up
Bao 2022 [98]	miR-519b-3p/PRRG4	Endometrial stromal cells Ectopic endometrium Eutopic endometrium	N/A	Up
Bendifallah 2022 [99]	hsa-miR-29b-1-5p hsa-miR-4748 hsa-miR-515-5p hsa-miR-548j-5p hsa-miR-6502-5p	Serum	I–IV	Up
Dabi 2022 [100]	miR-124-3p	Endometrial stromal cells Ectopic endometrium Eutopic endometrium Plasma	N/A	Up
Huang 2022 [102]	miR-301a-3p/PI3K	Ectopic endometrium Normal serum	N/A	Up
Iurova 2022 [103]	miR-92b-5p miR-4732-5p miR-3184-3p miR-423-5p miR-486-5p	Plasma	III–IV	Up
Jaafar 2022 [104]	miR-27a rs895819 miR-423 rs6505162	Serum	I–IV	Up

ASRM: American Society for Reproductive Medicine; N/A: not available.

**Table 3:** Downregulated microRNA expression profiles in patients with endometriosis.

Author, Year of Publication	MicroRNAs	Source	ASRM Stage of Disease	Regulation in Endometriosis
Laudanski 2013 [65]	hsa-miR-483-5p hsa-miR-629	Eutopic endometrium	III–IV	Down
Jia 2013 [6]	miR-17-5p miR-20a miR-22	Serum	III–IV	Down
Wang 2013 [66]	miR-145 miR-141 miR-542-3p	Serum	III–IV	Down
Cho 2015 [69]	miR-let-7a–f miR-135a/b	Serum	III–IV	Down
Rekker 2015 [7]	miR-200a-3p miR-200b-3p miR-141-3p	Plasma	I–IV	Down
Cosar 2016 [70]	miR-3613-5p	Serum	III–IV	Down
Wang 2016 [71]	miR-30c-5p miR127-3p miR-99b-5p miR-15b-5p	Serum	I–II	Down
Xu 2017 [73]	miR-3935 miR-4427 miR-652-5p miR-205-5p	Eutopic endometrium	N/A	Down
Bashti 2018 [21]	miR-31	Plasma	I–IV	Down
Liu 2018 [74]	miR-449b-3p	Endometrial stromal cells Ectopic endometrium Eutopic endometrium	I–II	Down
Pateisky 2018 [76]	miR-154-5p miR-196b-5p miR-378a-3p	Serum	I–IV	Down
Wang 2018 [77]	miR-17	Serum	I–IV	Down
Li 2019 [78]	miR-451	Follicular fluid	III–IV	Down
Petracco 2019 [80]	miR-135a/b	Ectopic endometrium Eutopic endometrium	II–III	Down
Zhang 2019 [82]	miR-141-5p	Ectopic endometrium Eutopic endometrium	III–IV	Down
Borisov 2020 [83]	miR-10b miR-200c	Eutopic endometrium	N/A	Down
Da Silva 2020 [84]	miR-532-3p	Cumulus cells	I–IV	Down
Gu 2020 [85]	let-7a-5p let-7b-5p let-7d-5p let-7f-5p let-7g-5p let-7i-5p miR-199a3p miR-320a miR-320b miR-320c miR-320d miR-328-3p miR-331-3p miR320e	Serum	III–IV	Down
Moustafa 2020 [87]	miR-3613-5p let-7b	Ectopic endometrium	I–IV	Down
Papari 2020 [88]	miR-17-5p miR-20a-5p miR-199a-3p miR-143-3p let-7b-5p	Plasma	III–IV	Down
Cui 2021 [92]	miR-9-5p	Endometrial stromal cells Ectopic endometrium Eutopic endometrium	III–IV	Down
Misir 2021 [93]	miR-34a-5p	Serum	I–IV	Down
Pokrovenko 2021 [94]	miR-let-7	N/A	I–IV	Down
Wang 2021 [95]	miR-205-5p/ZEB1	Ectopic endometrium Eutopic endometrium	III–IV	Down
Wu 2021 [96]	miR-15a-5p	Endometrial stromal cells Ectopic endometrium Eutopic endometrium	III–IV	Down
Zafari 2021 [97]	miR-224-5p miR let-7d-3p	Serum	I–IV	Down
Bendifallah 2022 [99]	hsa-miR-3137 hsa-miR-3168	Serum	I–IV	Down
He 2022 [101]	miR-148a	Serum	I–IV	Down
Huang 2022 [102]	miR-301a-3p/PTEN	Ectopic endometrium Normal serum	N/A	Down
Nai 2022 [105]	miR-363	Endometrial stromal cells Ectopic endometrium Eutopic endometrium	N/A	Down

ASRM: American Society for Reproductive Medicine; N/A: not available.

**Table 4:** MicroRNA modulation in endometriosis.

Author, Year of Publication	ASRM Stage of Disease	MicroRNAs	Dysmenorrhea (%)	Infertility (%)	Delta Patients vs. Controls
Laudanski 2013 [65]	III–IV	hsa-miR-483-5p hsa-miR-629	42.8	N/A	0.5 0.3
Jia 2013 [6]	III–IV	miR-17-5p miR-20a miR-22	N/A	21.7	N/A
Wang 2013 [66]	III–IV	miR-17-5p miR-20a miR-22 miR-199a miR-122	60.0	88.0	N/A
	III–IV	miR-145 miR-141 miR-542-3p	60.0	88.0	N/A
Suryawanshi 2014 [67]	N/A	miR-16 miR-191 miR-195	N/A	N/A	2.8 4.8 4.0
Braza-Boïls 2015 [68]	III–IV	miR-16-5p miR-21-5p miR-29c-3p miR-106b-5p miR-130a-5p miR-185-5p miR-195-5p miR-424-5p	N/A	N/A	0.1 6.8 3.0 4.8 0.7 1.0 7.7 1.2
Cho 2015 [69]	III–IV	miR-let-7a–f miR-135a/b	N/A	N/A	N/A
Rekker 2015 [7]	I–IV	miR-200a-3p miR-200b-3p miR-141-3p	57.3	63.9	0.6 0.5 0.7
Cosar 2016 [70]	III–IV	miR-125b-5pmiR-451a	N/A	N/A	0.1 0.4
	III–IV	miR-3613-5p	N/A	N/A	0.2
Wang 2016 [71]	I–II	miR-30c-5p miR127-3p miR-99b-5p miR-15b-5p miR-20a-5p	66	43	N/A
Nothnik 2017 [72]	I–IV	miR-451a	N/A	N/A	5.2
Xu 2017 [73]	N/A	miR-1304-3p miR-544b miR-3684 miR-494-5p miR-4683 miR-6747-3p	N/A	100	N/A
	N/A	miR-3935 miR-4427 miR-652-5p miR-205-5p	N/A	100	N/A
Bashti 2018 [21]	I–IV	miR-145	0.0	0.0	6.5
	I–IV	miR-31	0.0	0.0	0.95
Liu 2018 [74]	I–II	miR-449b-3p	N/A	N/A	0.5
Maged 2018 [75]	I–IV	miR-199 miR-122	53.3	N/A	N/A
Pateisky 2018 [76]	I–IV	miR-154-5p miR-196b-5p miR-33a-5p miR-378a-3p	N/A	N/A	N/A
Wang 2018 [77]	I–IV	miR-17	N/A	N/A	N/A
Li 2019 [78]	III–IV	miR-451	N/A	100	2.2
Nabiel 2019 [79]	I–IV	miR-17-5p	N/A	N/A	1.5
Petracco 2019 [80]	II–III	miR-135a/b	N/A	69.5	0.52
Vanhie 2019 [81]	I–IV	miR-125b-5p miR28-5p miR29a-3p	N/A	N/A	N/A
Zhang 2019 [82]	III–IV	miR-141-5p	N/A	N/A	0.75
Borisov 2020 [83]	N/A	miR-191	N/A	N/A	35
	N/A	miR-10b miR-200c	N/A	N/A	2.6 4
Da Silva 2020 [84]	I–IV	miR-532-3p	N/A	100	0.7
Gu 2020 [85]	III–IV	hsa-let-7a-5p hsa-let-7b-5p hsa-let-7d-5p hsa-let-7f-5p hsa-let-7g-5p hsa-let-7i-5p miR-199a3p miR-320a miR-320b miR-320c miR-320d miR-328-3p miR-331-3p miR320e	N/A	N/A	N/A
Mai 2020 [86]	III–IV	miR-506-5p	N/A	N/A	N/A
Moustafa 2020 [87]	I–IV	miR125b-5p miR-150-5p miR-342-3p miR-451a	N/A	N/A	N/A
	I–IV	miR-3613-5p let-7b	N/A	N/A	N/A
Papari 2020 [88]	III–IV	miR-17-5p miR-20a-5p miR-199a-3p miR-143-3p let-7b-5p	44.0	N/A	0.2 4.5 2.1 0.1 0.5
Hossein Razi 2020 [89]	III–IV	miR-185-5p	N/A	N/A	0.04
Wu 2020 [90]	III–IV	miR-423-5p	N/A	N/A	0.3
	III–IV	miR-214-3p	N/A	N/A	0.7
Zhang 2020 [91]	III–IV	miR-202-3p	N/A	N/A	0.3
Cui 2021 [92]	III–IV	miR-9-5p	N/A	N/A	0.7
Farsimadan 2021 [20]	N/A	miR-146a rs2910164 miR-149 rs2292832	N/A	100	2.2 2.9
Misir 2021 [93]	I–IV	miR-34a-5p miR-200c	73.2	94.1	N/A
Pokrovenko 2021 [94]	I–IV	miR-let-7	100	100	0.6
Wang 2021 [95]	III–IV	miR-205-5p/ZEB1	N/A	N/A	2.6
Wu 2021 [96]	III–IV	miR-15a-5p	N/A	0.0	13
Zafari 2021 [97]	I–IV	miR-199b-3p miR-224-5p miR let-7d-3p	N/A	N/A	N/A
Bao 2022 [98]	N/A	miR-519b-3p/PRRG4	N/A	N/A	3.37
Bendifallah 2022 [99]	I–IV	hsa-miR-29b-1-5p hsa-miR-4748 hsa-miR-515-5p hsa-miR-548j-5p hsa-miR-6502-5p	100	N/A	N/A
	I–IV	hsa-miR-3137 hsa-miR-3168	100	N/A	N/A
Dabi 2022 [100]	N/A	miR-124-3p	100	N/A	N/A
He 2022 [101]	I–IV	miR-148a	0.0	0.0	0.2
Huang 2022 [102]	N/A	miR-301a-3p/PI3K	N/A	N/A	0.2
	N/A	miR-301a-3p/PTEN	N/A	N/A	0.57
Iurova 2022 [103]	III–IV	miR-92b-5p miR-4732-5p miR-3184-3p miR-423-5p miR-486-5p	N/A	N/A	N/A
Jaafar 2022 [104]	I–IV	miR-27a rs895819 miR-423 rs6505162	N/A	100	0.9 2.4
Nai 2022 [105]	N/A	miR-363	N/A	N/A	0.01

ASRM: American Society for Reproductive Medicine; N/A: not available.

## Data Availability

No new data were created. Please see the References section for research data supporting reported results.
